# Correlation Between Triglyceride‐Glucose Index and Microvascular Complications in Patients With Early‐ Onset of Type 2 Diabetes Mellitus

**DOI:** 10.1002/edm2.70027

**Published:** 2025-02-13

**Authors:** Liu Ran, Yang Han, Hao Zhaohu, Shao Hailin

**Affiliations:** ^1^ The Tianjin Fourth Central Hospital of Tianjin Medical University Tianjin China; ^2^ Department of Cardiology, Tianjin Union Medical Center Nankai University Affiliated Hospital Tianjin China

**Keywords:** diabetic nephropathy, early‐onset diabetes mellitus, triglyceride–glucose index

## Abstract

**Objective:**

This study aimed to explore the potential correlation between the triglyceride‐glucose (TyG) index and diabetic nephropathy (DN) and diabetic retinopathy (DR) in patients with early‐onset type 2 diabetes mellitus (T2DM).

**Design:**

This cross‐sectional study statistically analysed TyG index levels across DN and DR stages in patients with early‐onset and non‐early‐onset T2DM.

**Patients:**

A total of 1530 T2DM patients were enrolled between January 2017 and August 2023 at Tianjin Fourth Central Hospital in Tianjin.

**Measurements:**

Correlation analysis and logistic regression were used to examine the association between the TyG index and microvascular complications. Kaplan–Meier plots and Cox regression analyses were employed to evaluate the effects of the TyG index on DN incidence. TyG index's diagnostic ability for DN was explored using the area under the receiver operating characteristic curve.

**Results:**

In patients with early‐onset T2DM, the TyG index gradually decreased with DR aggravation and gradually increased with DN aggravation, showing a negative correlation with DR and a positive correlation with DN in patients with early‐onset T2DM; logistic regression analysis suggested that the TyG index was an independent risk factor for DN (OR = 1.623, 95% CI = 1.175–2.242). The Cox regression analysis and Kaplan–Meier plots suggested that higher TyG was associated with an earlier incidence of DN in patients with early‐onset T2DM.

**Conclusion:**

In patients with early‐onset T2DM, the TyG index could be used to evaluate the risk of microvascular complications, with elevated TyG levels potentially indicating high risk of insulin– resistance related renal injury.

## Graphical abstract

In patients with early‐onset T2DM, the TyG index was associated with an earlier incidence of DN. The TyG index could be used to evaluate the risk of microvascular complications, elevated TyG levels potentially indicating high risk of insulin–resistance related renal injury.

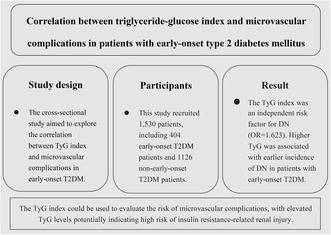

## 1 Introduction

As type 2 diabetes mellitus (T2DM) prevalence rises annually, diagnoses increasingly occur at younger ages [1, 2, 3]. Early‐onset T2DM is defined as a diagnosis of T2DM before the age of 40 years [4]. Patients with early‐onset T2DM have a higher and earlier incidence of diabetic nephropathy (DN) and diabetic retinopathy (DR) [5, 6], significantly affecting quality of life. Therefore, early detection and treatment of diabetic complications are crucial for patients with early‐onset T2DM. Diagnosing DN and DR typically requires the measurement of urinary albumin/creatinine ratio (UACR) and fundus examinations; however, these complex procedures can delay diagnosis, causing optimal treatment windows to be missed. Previous studies have shown that the triglyceride glucose (TyG) index, an accessible and cost‐effective biomarker for insulin resistance (IR), is linked to the development of diabetic microvascular complications [7, 8]. High burden of inflammation is an important reason for the aggravation of T2DM [9, 10] and diabetic microvascular complications [11, 12]; the TyG index is closely associated with inflammatory factors, such as the C‐reactive protein (CRP), which affect the inflammatory condition of the body [13]. However, the correlation of the TyG index with microvascular complications in patients with early‐onset T2DM remains unclear. This study aimed to establish the association between the TyG index and DN and DR in patients with early‐onset T2DM and explore its potential as a biomarker for microvascular complications in this population.

## 2 Materials and Methods

### 2.1 Study Participants

This retrospective study recruited 1530 patients with T2DM, enrolled between January 2017 and August 2023 at Tianjin Fourth Central Hospital in Tianjin, China. Inclusion criteria were: (1) participants with T2DM diagnosis according to the 1999 World Health Organisation (WHO) criteria and (2) age ≥ 18 years. Exclusion criteria were: (1) patients with severe dysfunction of the heart, liver, kidneys, or other organs; (2) patients with tumours; (3) patients who declined to provide informed consent; (4) patients with type 1 diabetes, other specific types of diabetes, or gestational diabetes; (5) patients with acute complications such as ketosis or hyperglycemic hyperosmolar state; (6) patients with co‐infections or other stress states. This study was approved by the Ethics Committee of Tianjin Fourth Central Hospital, and all participants provided informed consent.

### 2.2 Research Methods

Information was collected from medical records, including clinical information (age, sex, height, weight disease duration), body mass index (BMI; kg/m2), systolic blood pressure (SBP, mmHg), diastolic blood pressure (DBP, mmHg), fasting plasma glucose (FPG), serum creatinine (Cr), total cholesterol (TC), triglycerides (TG), glycosylated haemoglobin (HbAlc) and UACR (mg/g).

### 2.3 DR Diagnostic Criteria

All patients underwent pupil dilation by an ophthalmologist, followed by fundoscopy using a Heine fundoscope (MINI 3000; Germany). DR diagnosis was based on retinal abnormalities, including microaneurysms, haemorrhages, exudates and new vessel formats. The DR stages were classified using the 2002 International DR Severity Grading Standard into non‐diabetic retinopathy (NDR); mild, moderate or severe non‐proliferative diabetic retinopathy (NPDR); and proliferative diabetic retinopathy (PDR).

### 2.4 DN Diagnostic Criteria

According to the guidelines of the Global Kidney Disease Outcomes Improvement Organisation (KDIGO), DN was defined as a condition that meets all three of the following criteria: (1) T2DM diagnosis; (2) two consecutive urine tests within 6 months, demonstrating UACR ≥ 30 mg/g; (3) absence of other kidney diseases. DN stages were defined as normal albuminuria (UACR, < 30 mg/g), microalbuminuria (UACR, 30–300 mg/g) and macroalbuminuria (UACR, > 300 mg/g).

The TyG index was calculated as ln [(TG (mg/dl) × FPG (mg/dl))/2]. Renal function was assessed through estimated glomerular filtration rate (eGFR) using the Japanese Society of Nephrology's Chronic Kidney Disease Initiative formula: eGFR [ml/(min·1.73m^2^)] = 194 × serum creatinine—1.094 × age—0.287 (female × 0.739).

### 2.5 Statistical Analysis

Statistical analysis was performed using IBM SPSS Statistics for Windows, Version 22.0. Continuous variables were presented as mean ± standard deviation for normally distributed data and as median (interquartile range) otherwise. ANOVA was used to compare normally distributed variables between groups, and the Kruskal–Wallis H test was used to analyse non‐normally distributed data. The Spearman correlation analysis was used to test correlations between non‐normally distributed data. Cox regression analysis and Kaplan–Meier plots were performed to investigate the association between the TyG index and DN onset. Multivariate logistic regression was utilised to analyse the risk factors associated with DN. Receiver operating characteristic (ROC) curves, and the area under the curve (AUC) were used to assess the predictive value of the TyG index. Significance was set at *p* < 0.05.

## 3 Results

1. The study involved 1530 cases of individuals diagnosed with T2DM, divided into early‐onset (onset age < 40 years, 404 cases) and non‐early‐onset (onset age ≥ 40 years, 1126 cases) TDM groups. In the early‐onset T2DM group, BMI, T2DM duration, fasting blood glucose, glycosylated haemoglobin, diastolic blood pressure, triglycerides, total cholesterol, eGFR and TyG index were significantly higher compared with those of the non‐early‐onset T2DM group (*p* < 0.05), whereas age and systolic blood pressure were lower (*p* < 0.05). The basic information for both groups is presented in Table 1.

**TABLE 1:** Clinical information of participants in early‐onset and non‐early‐onset T2DM groups.

	Early‐onset T2DM	Non‐early‐onset T2DM	*p*
Number of participants	404	1126	
Sex (male/female)	292/112	621/505	
Age (years, M(P25, P75))	41 (36, 47)	60 (55, 64)*	0.000
BMI (kg/m2, M(P25, P75))	27.9 (25.5, 31.2)	26.7 (24.6, 29.0)*	0.000
Disease duration (year, M (P25, P75))	6 (5, 13)	5 (3, 7)*	0.000
Fasting blood glucose (mmol/L, M (P25, P75))	10 (8.3, 13)	9.3 (7.8, 11.3)*	0.000
Glycosylated haemoglobin (%, M(P25, P75))	9.2 (7.9, 10.7)	8.2 (7.3, 9.7)*	0.000
Systolic pressure (mmHg, M (P25, P75))	135 (124, 147)	142 (129, 155)*	0.000
Diastolic pressure (mmHg, M (P25, P75))	83 (75, 91)	80 (74, 88)*	0.000
Triglyceride (mmol/L, M (P25, P75))	2.0 (1.3, 3.2)	1.7 (1.2, 2.3)*	0.000
Total cholesterol (mmol/L, M (P25, P75))	5.2 (4.3, 6.0)	5.0 (4.3, 5.9)	0.278
eGFR (ml· min^−1^·1.73 m^−2^, M(P25, P75))	162.1 (131.5, 200.9)	119.6 (99.4, 143.6)*	0.000
TyG index M (P25, P75)	8.2 (7.6, 8.7)	7.9 (7.5, 8.3)*	0.000
ACR (mg/g, M(P25, P75))	3.4 (3.4, 15.4)	3.4 (3.4, 6.8)	0.134

*Note:* 1 mmHg = 0.133 kPa. *p < 0.05 compared with the early‐onset T2DM group.

Abbreviations: ACR, the urinary albumin/creatinine ratio; BMI, body mass index; eGFR, estimated glomerular filtration rate; T2DM, type 2 diabetes mellitus; TyG, triglyceride‐glucose.

2. Patients with early‐onset and non‐early‐onset T2DM were further stratified based on DN and DR severity. TyG index values were statistically analysed for each group. In the early‐onset T2DM group, TyG index values decreased with increasing DR severity; and they were significantly lower in patients with moderate to severe NPDR than in those with NDR (Table 2a) (*p* < 0.05). TyG index values increased with DN severity in the early‐onset T2DM group (Table 2b), with significantly higher levels in patients with micro/macro‐albuminuria than in those with normal albuminuria (*p* < 0.05). In the non‐early‐onset T2DM group, the TyG index level increased significantly with DN severity (Table 2b) (*p* < 0.05); however, it did not differ significantly by DR stage (*p* > 0.05).

**TABLE 2a:** Comparison of TyG index levels in early‐onset/ non‐early‐onset T2DM group with different severity of DR.

	NDR	Mild NPDR	Moderate and severe NPDR and PDR
Early‐onset T2DM group			
*N*	320	33	51
TyG index M(P25, P75)	8.25 (7.67, 8.73)	8.18 (7.76, 8.57)	7.88 (7.39, 8.45)*
Non‐early‐onset T2DM group			
*N*	1026	43	57
TyG index M(P25, P75)	7.87 (7.48, 8.27)	7.79 (7.41, 8.54)	7.83 (7.39, 8.31)

*Note:* *p < 0.05 compared with the NDR group.

Abbreviations: DR, diabetic retinopathy; NDR, non‐diabetic retinopathy; NPDR, non‐proliferative diabetic retinopathy; PDR, proliferative diabetic retinopathy; T2DM, type 2 diabetes mellitus; TyG, triglyceride‐glucose.

**TABLE 2b:** Comparison of TyG index levels in early‐onset/ non‐early‐onset T2DM groups with different severity of DN.

	Normal albuminuria	Microalbuminuria and macroalbuminuria
Early‐onset T2DM group		
*N*	318	86
TyG index M(P25, P75)	8.14 (7.58, 8.55)	8.47 (7.84, 9.10)*
Non‐early‐onset T2DM group		
*N*	915	211
TyG index M(P25, P75)	7.85 (7.43, 8.26)	8.03 (7.63, 8.40)*

*Note:* *p < 0.05 compared with the normal albuminuria group.

Abbreviations: DN, diabetic nephropathy; T2DM, type 2 diabetes mellitus; TyG, triglyceride‐glucose.

3. Spearman correlation analysis revealed that in the early‐onset T2DM group, TyG index values were negatively correlated with DR severity (*R* = −0.122, *p* < 0.05) and positively correlated with DN severity (*R* = 0.149 *p* < 0.05). TyG index values were positively correlated with DN severity (*R* = 0.105, *p* < 0.05); however, no significant correlation was observed with DR in the non‐early‐onset T2DM group (*p* > 0.05) (Table 3).

**TABLE 3:** Correlation analysis between the TyG index and the severity of DR and DN.

Early‐onset T2DM group		Non‐early‐onset T2DM group	
DR			
*R*	−0.122	R	−0.015
*p*	0.014	P	0.613
DN			
*R*	0.149	R	0.105
*p*	0.003	P	0.000

Abbreviations: DN, diabetic nephropathy; DR, diabetic retinopathy; T2DM, type 2 diabetes mellitus; TyG, triglyceride‐glucose.

**FIGURE 1:**
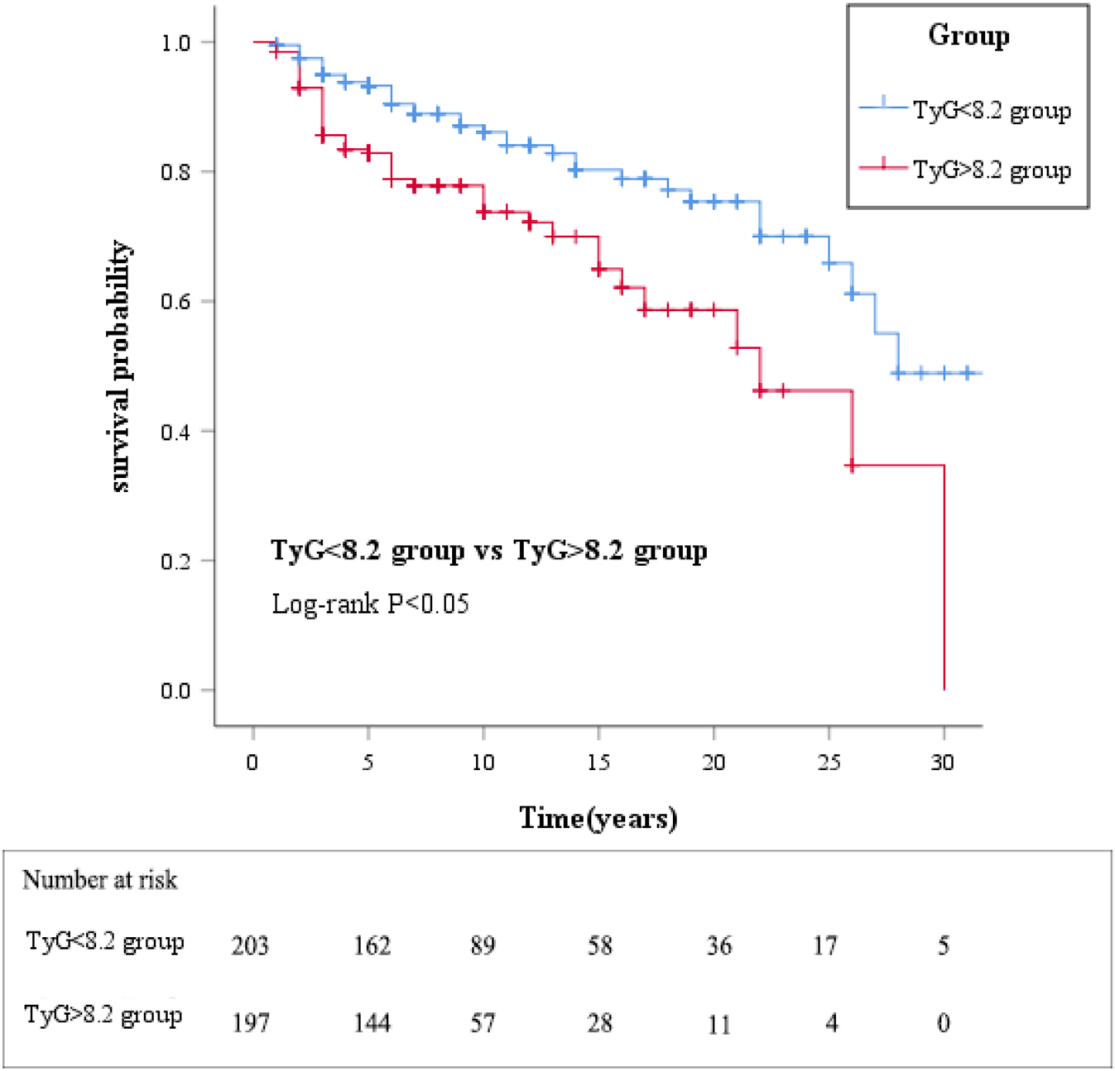
Kaplan–Meier analysis of DN disease‐free survival with different levels of the TyG. Index in the early‐onset T2DM group. Kaplan–Meier curves comparing disease‐free survival for DN between individuals with TyG < 8.2 and individuals with TyG > 8.2 (Log‐Rank test: *p* < 0.05). DN, Ddiabetic nephropathy; T2DM, Ttype 2 diabetes mellitus; TyG, Ttriglyceride‐glucose.

4. COX regression analysis revealed that in the early‐onset T2DM group, the TyG index (hazard ratio [HR] = 1.400, 95% confidence interval [CI]: 1.076–1.823), age (HR = 0.937, 95% CI: 0.910–0.966), BMI (HR = 1.051, 95% CI: 1.004–1.100) and diastolic blood pressure (HR = 1.046, 95% CI: 1.016–1.076) were significantly correlated with DN incidence (*p* < 0.05). The Kaplan–Meier analysis, grouped by the TyG index median, showed significantly higher cumulative DN incidence in patients with a higher TyG index (Figure 1) (Log‐rank *p* < 0.05). In the non‐early‐onset T2DM group, age (HR = 0.975, 95% CI: 0.953–0.998), BMI (HR = 1.045, 95% CI: 1.011–1.080) and diastolic blood pressure (HR = 1.020, 95% CI: 1.005–1.035) correlated significantly with DN incidence (*p* < 0.05), while the TyG index was not significantly correlated with DN incidence (Table 4).

**TABLE 4:** Cox regression analysis of factors associated with DN incidence.

	Non‐early‐onset T2DM group				Early‐onset T2DM group			
	HR	95% CI		*p*	HR	95% CI		*p*
Sex	1.248	0.947	1.646	0.116	0.866	0.528	1.422	0.571
Age	0.975	0.953	0.998	0.030*	0.937	0.910	0.966	0.000*
BMI	1.045	1.011	1.080	0.009*	1.051	1.004	1.100	0.035*
Diastolic pressure	1.020	1.005	1.035	0.008*	1.046	1.016	1.076	0.002*
Systolic pressure	1.008	0.999	1.017	0.071	0.990	0.971	1.009	0.306
TyG Index	1.119	0.901	1.391	0.309	1.400	1.076	1.823	0.012*

*Note:* *p < 0.05 indicates significant correlation with DN incidence.

Abbreviations: BMI, body mass index; CI, confidence interval; DN, diabetic nephropathy; HR, hazard ratio; T2DM, type 2 diabetes mellitus; TyG, triglyceride‐glucose.

5. Multivariable logistic regression analysis showed that in early‐onset T2DM, the TyG index (odds ratio [OR] = 1.623, 95% CI: 1.175–2.242), disease duration (OR = 0.929, 95% CI: 0.873–0.988) and systolic blood pressure (OR = 1.025, 95% CI 1.003–1.048) were independent risk factors for DN (*p* < 0.05). In non‐early‐onset T2DM, BMI (OR = 1.046, 95% CI: 1.006–1.092), systolic blood pressure (OR = 1.225, 95% CI: 1.003–1.023), and disease duration (OR = 1.013, 95% CI: 0.895–0.973) were independent risk factors for DN (*p* < 0.05), while the TyG index was not an independent risk factor for DN (Table 5).

**TABLE 5:** Logistic regression analysis of risk factors associated with DN.

	Non‐early‐onset T2DM group					Early‐onset T2DM group				
	B	*p*	95% CI		OR	B	*p*	95% CI		OR
Sex	0.173	0.281	0.868	1.628	1.213	−0.163	0.585	0.473	1.526	0.849
Age	0.008	0.504	0.984	1.033	1.011	0.044	0.063	0.998	1.095	1.045
BMI	0.047	0.026*	1.006	1.092	1.046	0.006	0.822	0.953	1.063	1.006
Diastolic pressure	0.014	0.114	0.997	1.033	0.938	0.007	0.677	0.975	1.039	1.007
Systolic pressure	0.013	0.013*	1.003	1.023	1.225	0.025	0.027*	1.003	1.048	1.025
Disease duration	−0.069	0.001*	0.895	0.973	1.013	−0.074	0.020*	0.873	0.988	0.929
TyG Index	0.233	0.065	0.986	1.618	1.013	0.484	0.003*	1.175	2.242	1.623

*Note:* *p < 0.05; indicates independent risk factors for DN.

Abbreviations: BMI, body mass index; DN, diabetic nephropathy; T2DM, type 2 diabetes mellitus; TyG, triglyceride‐glucose.

6. The area under the ROC curve (AUC) for DN was 0.603 (0.531–0.675) in the early‐onset T2DM group and 0.564 (0.522–0.606) in the non‐early‐onset T2DM group, indicating low discrimination ability for DN when using the TyG index as an independent indicator.

## 4 Discussion

With the increasing prevalence of T2DM, the population of patients with early‐onset T2DM continues to expand. Although early‐onset and non‐early‐onset T2DM share similar pathological mechanisms, early‐onset T2DM exhibits more severe insulin resistance and greater susceptibility to both macrovascular and microvascular complications. Research indicates that patients with early‐onset T2DM have a significantly higher DN, DR and peripheral neuropathy incidence compared to those with type 1 diabetes [14]. In a cohort study by Li et al. [15] involving 29,442 patients with T2DM, DN and DR incidence in patients with early‐onset T2DM was 5.1% and 7.1%, respectively, much higher than those in patients with non‐early‐onset T2DM (1.5% and 2.7%). Currently, DN and DR diagnosis relies on UACR measurement and fundus examination, which are complex to operate, with difficulties in collecting/preserving specimens; consequently, optimal diagnostic and treatment opportunities may potentially be missed. The TyG index, known for its cost‐efficiency and simplicity, serves as an accessible indicator of insulin resistance. This study's results demonstrated that although the AUC for the TyG index in diagnosing DN and DR was < 0.7, the TyG index was significantly correlated with DN and DR in patients with early‐onset T2DM and showed substantial changes as diabetic microvascular disease worsened. These findings suggest that while the TyG index may not independently diagnose diabetic microvascular complications, regular monitoring and observation of its changes can help evaluate complication severity in patients with early‐onset T2DM.

DN is a common microvascular complication of T2DM and the leading cause of renal failure in patients with diabetes. Previous studies suggest that DN results from prolonged hyperglycemia, which damages the glomerular filtration membrane, causes inflammation and leads to fibrosis, lowering the GFR and impairing renal tubular function. While DN prevalence is higher and develops faster in individuals with early‐onset T2DM, no research exists on the differences in DN pathogenesis between early‐onset and non‐early‐onset T2DM. This study found a significant correlation between the TyG index and DN in both early‐onset and non‐early‐onset T2DM, with a stronger correlation in the early‐onset T2DM population. As DN progresses, the TyG index gradually increases. The regression analysis suggests that a higher TyG index is associated with earlier DN incidence in early‐onset T2DM. Since the TyG index reflects insulin resistance severity, these results imply that kidney outcomes in patients with early‐onset T2DM are closely linked to insulin resistance severity. Previous studies indicate that patients with early‐onset T2DM experience more severe insulin resistance and hyperinsulinemia than those with non‐early‐onset T2DM. Hyperinsulinemia can cause renal vasodilation, increased permeability, elevated glomerular hydrostatic pressure and high glomerular filtration [16, 17] Excessive filtration load leads to the loss of nephrons, which in turn causes abnormal renal function [18]. Additionally, many inflammatory and metabolic markers are associated with diabetic kidney injury; notably, the CRP [19], kidney injury molecule [20], omentin [21], mean platelet volume [22], serum uric acid [23], monocyte/lymphocyte ratio in hemogram [24], systemic inflammatory index [25], neuregulin [26] and uric acid [27]. Obesity and insulin resistance can activate inflammatory factors in peripheral tissues, contributing to renal damage through inflammation and oxidative stress [28, 29]. Therefore, we speculate that severe insulin resistance, amongst other factors, contributes to early renal injury in early‐onset T2DM. In summary, insulin resistance is a primary factor in renal injury in individuals with early‐onset T2DM; however, the specific mechanisms require further studies. Regular monitoring of the TyG index in early‐onset T2DM patients may assist in assessing insulin– resistance related renal injury.

DR is another common T2DM complication. This study's results showed that in the early‐onset T2DM group, the TyG index was negatively correlated with DR severity and displayed a gradual decline as DR progressed. Previous studies have explored the relationship between the TyG index and DR; however, findings have been inconsistent. For instance, a meta‐analysis by Zhou et al. found that patients with higher TyG index values were more likely to have DR [30], while Yao et al. found in a comparative study that the TyG index was lower in patients with DR and decreased as DR severity increased [31]; why do these studies reach different conclusions? The previous studies did not differentiate between patients with early‐onset and non‐early‐onset T2DM. As this study reveals, the relationship between the TyG index and DR varies between early‐onset and non‐early‐onset T2DM, and differences in study populations likely led to variations in research results. Most of the previous research suggests that insulin resistance and high glucose levels jointly contribute to DR, while insulin resistance directly promotes the deterioration of DR [32, 33]. Rithwick et al. observed in animal studies that high‐fat‐fed mice exhibited activation of retinal stress kinase and neuroinflammatory cells before blood sugar abnormalities appeared. However, this study found that insulin resistance in early‐onset T2DM patients was negatively correlated with DR severity. In previous studies, the homeostatic model assessment for insulin resistance (HOMA‐IR) was usually used as the biomarker of insulin resistance severity. HOMA‐IR is calculated from the FPG and insulin, reflecting the metabolic status of glucose and insulin, while the TyG index is calculated from the FPG and TG, reflecting the combined effect of glucose and lipids. Thus, the relationship between the TyG index and DR may not be the same as that between HOMA‐IR and DR. Furthermore, different characteristics and eating habits of the individuals in these studies may have also led to the difference in results. Nonetheless, the difference between the TyG index and HOMA‐IR still needs further investigation.

Our study results demonstrated that the AUC for the TyG index in diagnosing DN and DR was < 0.7, showing weak performance as an independent diagnostic tool. Nonetheless, it is worth investigating whether combining the TyG index with other biomarkers enhances its utility. Xu C et al. [34] showed that the AUC of the TyG‐waist‐to‐height ratio and TyG‐waist circumference is higher than that of the TyG index in diagnosing sarcopenic obesity. In a study conducted by Mu X et al. [35], a prediction model for DN was constructed based on the TyG index, low‐density lipoprotein (LDL), high‐density lipoprotein (HDL) and 37 other indicators, achieving an AUC of 0.826. We will further investigate the predictive ability of TyG‐waist‐to‐height ratio and TyG‐waist circumference for early‐onset‐T2DM‐associated microvascular complications and construct a predictive model based on the TyG index with the aim of improving its diagnostic value.

Although DR and DN was significantly associated with the TyG index in early‐onset T2DM patients, the correlations were weak (*R* = −0.122 and 0.149), suggesting that other factors may be influencing the association between the TyG index and diabetic microvascular complications. To investigate the direct effect of the TyG index on DN, we included other factors to construct multiple regression models, reaching an OR up to 1.623 based on the TyG index. These results suggested a definite association between elevated TyG and the development of DN in early‐onset T2DM; nonetheless, elevated TyG index cannot be the only evidence for the occurrence of DN since many other factors affect the pathology of DN. In clinical work, elevated TyG index can only indicate elevated risk for early occurrence of DN. In order to improve the diagnostic efficiency, constructing a prediction model with multiple biomarkers is necessary.

This study had limitations: the number of patients with moderate to severe DR and macroalbuminuria was relatively small, which may have affected statistical accuracy. Additionally, the cross‐sectional observational design cannot establish causality between the TyG index and microvascular complications in patients with early‐onset T2DM. Future cohort studies with larger patient samples are needed to clarify the relationship between the TyG index and microvascular complications in patients with early‐onset T2DM. Additionally, the TyG index is directly calculated based on triglyceride and glucose values, and high triglyceride levels can directly cause kidney damage, independent of other factors. Renal mesangial cells can capture triglyceride‐rich lipid particles, which can induce kidney damage [36]. Triglycerides can also cause renal injury by inducing endothelial dysfunction and oxidative stress [37]. Therefore, using the TyG index to assess the impact of insulin resistance on DN may include the effects of direct kidney damage from hypertriglyceridemia. Future research will explore the TyG index's effectiveness in assessing microvascular disease by grouping patients according to triglyceride levels. Due to the limitation of experimental conditions, we could not obtain information about patients in other regions, thus, the generalisability of the findings still needs verification. In this cross‐sectional study, the association of the TyG index with microvascular complications was confirmed, but the sequential and causal relationship between the two requires further exploration. Longitudinal studies are necessary to access the development of TyG index and microvascular complications with the increasing disease duration in early‐onset type 2 diabetes patients, thus to clarify the causal relationship between them. We will continue to follow up eligible individuals to obtain longitudinal data and further explore the relationship between TyG and microvascular complications.

In summary, the progression of diabetic microvascular disease in patients with early‐onset T2DM is closely related to insulin resistance severity. The TyG index quickly reflects microvascular complication changes in early‐onset T2DM, and an elevated TyG index may indicate insulin– resistance related renal injury in patients with early‐onset T2DM.

## Author Contributions

All authors met the authorship requirements. Liu Ran has taken primary responsibility for statistical analyses and writing original draft. Yang Han has assisted in writing and editing for this research. Dr. Hao Zhaohu has provided valuable guidance for study design, reviewing and editing. Dr. Shao Hailin has been instrumental in collecting and organizing data, funding acquisition and supervision. All authors have contributed to the revision and refinement of the article, ensuring its clarity and accuracy. We would like to express our gratitude to each author for their contributions to this article.

## Conflicts of Interest

The authors declare no conflicts of interest.

## Data Availability

The data that support the findings of this study are available from the corresponding and first author upon reasonable request.
